# MAOB expression correlates with a favourable prognosis in prostate cancer, and its genetic variants are associated with the metastasis of the disease

**DOI:** 10.1111/jcmm.18229

**Published:** 2024-03-23

**Authors:** Hsiang‐Ching Huang, Yi‐Hsien Hsieh, Chi‐Hao Hsiao, Chia‐Yen Lin, Shian‐Shiang Wang, Kuo‐Hao Ho, Lun‐Ching Chang, Huei‐Mei Huang, Shun‐Fa Yang, Ming‐Hsien Chien

**Affiliations:** ^1^ Graduate Institute of Medical Sciences, College of Medicine Taipei Medical University Taipei Taiwan; ^2^ Institute of Medicine Chung Shan Medical University Taichung Taiwan; ^3^ Department of Medical Research Chung Shan Medical University Hospital Taichung Taiwan; ^4^ Department of Urology, School of Medicine, College of Medicine and TMU Research Center of Urology and Kidney (TMU‐RCUK) Taipei Medical University Taipei Taiwan; ^5^ Department of Urology, Wan Fang Hospital Taipei Medical University Taipei Taiwan; ^6^ Division of Urology, Department of Surgery Taichung Veterans General Hospital Taichung Taiwan; ^7^ School of Medicine Chung Shan Medical University Taichung Taiwan; ^8^ School of Medicine National Yang Ming Chiao Tung University Taipei Taiwan; ^9^ Department of Applied Chemistry National Chi Nan University Nantou Taiwan; ^10^ Graduate Institute of Clinical Medicine, College of Medicine Taipei Medical University Taipei Taiwan; ^11^ Department of Mathematical Sciences Florida Atlantic University Boca Raton Florida USA; ^12^ Pulmonary Research Center Wan Fang Hospital, Taipei Medical University Taipei Taiwan; ^13^ Traditional Herbal Medicine Research Center Taipei Medical University Hospital Taipei Taiwan; ^14^ TMU Research Center of Cancer Translational Medicine Taipei Medical University Taipei Taiwan

**Keywords:** clinicopathologic development, monoamine oxidase B, prognosis, prostate cancer, single‐nucleotide polymorphism

## Abstract

Monoamine oxidase B (MAOB), a neurotransmitter‐degrading enzyme, was reported to reveal conflicting roles in various cancers. However, the functional role of MAOB and impacts of its genetic variants on prostate cancer (PCa) is unknown. Herein, we genotyped four loci of MAOB single‐nucleotide polymorphisms (SNPs), including rs1799836 (A/G), rs3027452 (G/A), rs6651806 (A/C) and rs6324 (G/A) in 702 PCa Taiwanese patients. We discovered that PCa patients carrying the MAOB rs6324 A‐allele exhibited an increased risk of having a high initial prostate‐specific antigen (iPSA) level (>10 ng/mL). Additionally, patients with the rs3027452 A‐allele had a higher risk of developing distal metastasis, particularly in the subpopulation with high iPSA levels. In a subpopulation without postoperative biochemical recurrence, patients carrying the rs1799836 G‐allele had a higher risk of developing lymph node metastasis and recurrence compared to those carrying the A‐allele. Furthermore, genotype screening in PCa cell lines revealed that cells carrying the rs1799836 G‐allele expressed lower MAOB levels than those carrying the A‐allele. Functionally, overexpression and knockdown of MAOB in PCa cells respectively suppressed and enhanced cell motility and proliferation. In clinical observations, correlations of lower MAOB expression levels with higher Gleason scores, advanced clinical T stages, tumour metastasis, and poorer prognosis in PCa patients were noted. Our findings suggest that MAOB may act as a suppressor of PCa progression, and the rs3027452 and rs1799836 genetic variants of MAOB are linked to PCa metastasis within the Taiwanese population.

## 1 INTRODUCTION

Prostate cancer (PCa) has a high global incidence and ranks as the second most common cancer in men.^1^, ^2^ Since 2014, overall incidence rates of PCa have annually increased by 3%, with a 5% annual increase specifically in advanced stages.^3^ In Taiwan, there has been a significant rise in PCa incidence over the past decades, with 18.35% of patients diagnosed at Stage III and a remarkable 33% at Stage IV.^4^ Despite advancements in treating metastatic PCa, managing the disease remains challenging,^5^ resulting in a persistently high mortality rate.^6^ This is attributed, in part, to limited resources for PCa screening and detection. Until now, the conventional diagnostic approach for PCa has involved a prostate biopsy, often prompted by assessments of the prostate‐specific antigen (PSA) level in the blood or a digital rectal examination. However, limitations exist, as there are cases where PSA is insufficient,^7^ and numerous studies have highlighted the poor correlation of PSA levels with survival outcomes.^8^ Moreover, the prostate biopsy is an interventional procedure, emphasizing the need for new, more specific markers.

Recently, there has been growing interest in studying genetic risk factors, particularly single‐nucleotide polymorphisms (SNPs), for the early prediction and prognosis of PCa.^9^ SNPs refer to the substitution, insertion, or deletion of a single nucleotide at a specific genomic position, occurring in nearly 1 out of 800 base pairs.^10^ Such variants have a frequency in DNA of at least 1% in the general population. SNPs can cause variations in genes, altering the protein and enzymatic machinery of cells, ultimately impacting susceptibility to diseases and responses to treatment. Indeed, several genome‐wide association studies (GWASs) on PCa susceptibility in large cohorts reported the significance of hundreds of SNPs in relation to PCa incidence and progression.^11^ For example, Y‐box‐binding protein (YB)‐1 plays a crucial role in regulating androgen receptor (AR) variants, which are implicated in resistance to androgen deprivation therapy (ADT) for PCa.^12^ The intronic SNP rs1203072 of the *YB‐1* gene was reported to influence gene expression and correlate with PCa metastasis.^13^ In addition to YB‐1, SNPs located in genes such as solute carrier organic anion transporter family member 1B3 (SLCO1B3), SLCO2B1, caspase‐3 (CASP3), src kinase‐associated phosphoprotein 1 (SKAP1), and others were implicated in regulating androgen transporters, AR function and cell apoptosis. These SNPs were shown to be correlated with treatment outcomes of ADT in PCa.^14^

The monoamine oxidase (MAO) enzyme family, which includes MAOA and MAOB and is located on the X‐chromosome (Xp11.23), was initially recognized for catalysing the oxidative deamination of monoamine neurotransmitters in the nervous system and was associated with several neurological diseases.^15^ Recent studies revealed conflicting roles of MAOs in regulating tumour progression in various cancers. For instance, there was a significant increase in MAOA levels observed in glioblastoma multiforme and non‐small cell lung cancer compared to normal tissues. Additionally, the elevated MAOA level exhibited a positive correlation with clinical stages and lymph node metastasis.^16^, ^17^ In contrast, a positive correlation was reported between MAOA and favourable prognosis in patients with hepatocellular carcinoma, where MAOA was found to suppress HCC metastasis.^18^ Furthermore, in cholangiocarcinoma tissue, MAOA expression was downregulated through promoter hypermethylation. Notably, overexpression of MAOA demonstrated an inhibitory effect on cholangiocarcinoma growth and invasion.^19^ In PCa, MAOA was reported to induce the epithelial‐to‐mesenchymal transition (EMT)‐mediated metastasis of cells.^20^ MAOA expression was also correlated with resistance of PCa to chemotherapy (docetaxel)^21^ and ADT (enzalutamide).^22^ Although the clinical significance and functional role of MAOA in PCa were investigated by several studies, the impacts of MAOB on PCa remain poorly explored. In this study, our aim was to investigate the functional role of MAOB in PCa progression and further assess associations of SNPs within the MAOB gene with PCa clinicopathologic development.

## 2 MATERIALS AND METHODS

### 2.1 Study participants

In this study, we enrolled a cohort of 702 patients diagnosed with prostate adenocarcinoma who had undergone a robotic‐assisted laparoscopic radical prostatectomy (RP) with bilateral standard pelvic lymph node dissection between 2012 and 2018 at Taichung Veterans General Hospital in Taichung, Taiwan. Medical information pertaining to PCa patients at the time of diagnosis was collected from their medical records. This information included the pathologic Gleason grade, PSA values, clinical and pathologic TNM (tumour, node and metastasis) staging, D'Amico classification, and the extent of cancer cell invasion in areas such as the perineural, seminal vesicle and lymphovascular regions. The definition of biochemical recurrence (BCR) was based on the occurrence of two consecutive PSA values exceeding 0.2 ng/mL. Peripheral blood samples were collected from each participant after obtaining informed consent, and the study protocol was approved by the Institutional Review Board of Taichung Veterans General Hospital (IRB no. CE19062A).

### 2.2 Specimen collection and genomic DNA extraction

Peripheral blood was collected from all recruited PCa patients and preserved in EDTA‐containing anti‐coagulant tubes. Genomic DNA was subsequently extracted using a QIAamp DNA Blood Mini Kit (Qiagen, Valencia, CA, USA) following the manufacturer's instructions. Extracted DNA was dissolved in Tris‐EDTA (TE) buffer (10 mM Tris and 1 mM EDTA; pH 7.8), and the DNA purity was assessed with a Nanodrop‐2000 spectrophotometer (ThermoFisher Scientific, Waltham, MA, USA) to determine the ratio of absorbances at 260 and 280 nm. The final DNA preparation was stored at −20°C before undergoing a real‐time polymerase chain reaction (PCR) analysis.

### 2.3 Selection and determination of MAOB genetic polymorphisms

In total, four SNPs in MAOB, namely rs1799836 (A/G), rs3027452 (G/A), rs6651806 (A/C), and rs6324 (G/A), were selected for analysis. These specific SNPs were chosen based on previous reports indicating their correlations with the risk or progression of cancers or other diseases and their impacts on the activity or expression of MAOB.^23^, ^24^, ^25^, ^26^ Allelic discrimination of the MAOB rs1799836 (assay ID: C_8878790_10), rs3027452 (assay ID: C_15763403_10), rs6651806 (assay ID: C_29047318_10) and rs6324 (assay ID: C_11617922_10) SNPs was performed using a TaqMan SNP Genotyping Assay, utilizing the ABI StepOnePlus™ Real‐Time PCR System (ThermoFisher Scientific). Detailed procedures for DNA genotyping are described in our previously published study.^27^

### 2.4 Bioinformatics analysis

The UCSC Xena database (https://xena.ucsc.edu/) provided clinical data and messenger (m)RNA sequencing information for prostate adenocarcinoma (PRAD) samples from The Cancer Genome Atlas (TCGA). In this study, a Spearman correlation analysis was employed to identify gene candidates linked with expressions of the *MAOA* or *MAOB* genes. Genes displaying a Spearman correlation of >0.3 or <−0.3 were considered correlated with MAOA or MAOB. Subsequently, a Cox regression analysis was conducted to assess the prognostic impacts of MAOA‐ or MAOB‐associated genes. Genes exhibiting a hazard ratio of >1 and *p* < 0.05 were categorized as poor prognostic candidates, while those with a hazard ratio of <1 and *p* < 0.05 were classified as good prognostic candidates. The remaining genes were categorized as non‐prognostic candidates. We further conducted a comparative analysis of expression levels of the *MAOA* and *MAOB* genes between PRAD and normal tissues. Additionally, we assessed MAOB expression levels in relation to various clinical features, including clinical TNM stages and Gleason scores. For two‐group comparisons, the Wilcoxon signed‐rank test was employed, while the Kruskal–Wallis test with post hoc Dunn's test was utilized for clinical features with more than two groups. To evaluate the association between MAOB expression and patients' overall survival (OS), progression‐free survival (PFS) and disease‐specific survival (DSS), a log‐rank test was applied. High (MAOB^high^) and low (MAOB^low^) expression groups were determined based on the median cut‐off point of MAOB.

### 2.5 PCa cell lines and culture

In this study, we utilized a panel of cell lines with progressively increasing metastatic potential, as previously described.^28^ These cell lines included PC3M (highly aggressive), PC3 (moderately metastatic), LNCaP (lowly metastatic) and 22Rv1 (tumorigenic but not metastatic), all obtained from American Type Culture Collection (ATCC; Manassas, VA, USA). PC3 and PC3M cells were cultured in minimum essential medium (MEM, Life Technologies, Carlsbad, CA, USA), while LNCaP and 22Rv1 cells were maintained in RPMI‐1640 medium (Life Technologies). All culture media used in this study were supplemented with 10% fetal bovine serum (FBS, Gibco‐BRL, Gaithersburg, MD, USA) and 1% penicillin–streptomycin‐glutamine. PCa cells were maintained in an incubator at 37°C with a 5% CO_2_ and 95% air atmosphere.

### 2.6 Total protein extraction from PCa cells and western blot analysis

Protein lysates were extracted using a PRO‐PREPTM protein extraction solution (iNtRON Biotechnology, Seongnam, Korea), which contains a protease inhibitor cocktail as previously described.^29^ Total protein concentrations were determined using a Bio‐Rad protein assay kit (Bio‐Rad, Hercules, CA, USA). Subsequently, proteins (20–40 μg) were separated by sodium dodecylsulphate polyacrylamide gel electrophoresis (SDS‐PAGE) and electrophoretically transferred to polyvinylidene difluoride (PVDF) membranes (Bio‐Rad). The PVDF membranes were sequentially incubated with the primary MAOB antibody (Abcam, Cambridge, MA, USA) and its horseradish peroxidase‐conjugated secondary antibody (Santa Cruz Biotechnology, Santa Cruz, CA, USA). After being washed, membranes were incubated with an enhanced chemiluminescence (ECL) Western blotting reagent (Pierce Biotechnology, Rockford, IL, USA), and chemiluminescence was detected using the MultiGel‐21 chemiluminescence imaging system (TOP BIO, New Taipei City, Taiwan).

### 2.7 Construction of the Y435W mutation of MAOB

The pLX304‐MAOB plasmid was obtained from the DNASU repository (https://dnasu.org/DNASU/Home.do) and employed as a template. A MAOB‐Y435W mutation construct was generated using the QuickChange® Lightning Site‐directed Mutagenesis kit (Agilent Technology, La Jolla, CA, USA). A specific primer pair (5′‐CACTGGAGCGGCTGGATGGAGGGGGCTG‐3′ and 5′‐CAGCCCCCTCCATCCAGCCGCTCCAGTG‐3′) was used to replace the target sequence. Mutant DNA was amplified using Pfu Ultra II DNA polymerase, and template DNA was subsequently digested with DpnI endonuclease to remove non‐mutated plasmids. The plasmid harbouring the mutant *MAOB* gene was then transformed into *Escherichia coli* DH5α bacteria for amplification and isolation. Finally, the mutant gene was verified through sequencing.

### 2.8 Transfection and viral infection

Short hairpin (sh)MAOB constructs were obtained from the National RNAi Core Facility of Academia Sinica (Taipei, Taiwan). Target sequences for MAOB were 5′‐CCAGAATCGTATCTTGAGAT‐3′ (shRNA1) and 5′‐TAGGATTGGAGACCTACAAAG‐3′ (shRNA2). Co‐transfection of the shRNA or pLenti‐MAOB‐expressing vector (pLX304‐MAOB), envelope plasmid pMD, and pCMV‐ΔR8.91 into 293T cells was employed to produce shMAOB or MAOB‐overexpressing lentiviruses. Stable MAOB‐manipulated PCa cells were selected using puromycin (for knockdown) or blasticidin (for overexpression). To examine the effects of MAOB manipulation on cell behaviours, including migration and invasion, various cell behaviour assessments were performed within 72 h post‐transfection.

### 2.9 Cell viability and colony‐formation assays

PCa cells with manipulated MAOB expression were plated in 96‐well plates at a density of 5 × 10^3^ cells/well in culture medium containing 10% FBS. After 24 and 48 h, a cell viability assay (MTS assay; Promega, Madison, WI, USA) was conducted according to the manufacturer's instructions. Data were obtained from three independent replicates. For the colony‐forming assay, PC3M cells expressing MAOB or a control vector (1000 cells) were seeded in six‐well plates. The medium was changed every 2 days, and after 14 days of incubation, cells were stained with crystal violet. Colonies were manually counted using free ImageJ software (National Institutes of Health, Bethesda, MD, USA).

### 2.10 Transwell migration and invasion assays

Migration and invasion assays were conducted following the methodology outlined in our prior study.^30^ In brief, for the migration assay, 2 × 10^5^ PCa cells, either control or manipulated for MAOB expression, were seeded in an uncoated top chamber (24‐well insert; pore size, 8 μm; Corning Costar, Corning, NY, USA). In the invasion assay, 4 × 10^5^ cells were plated in the top chamber coated with Matrigel (BD Biosciences, Bedford, MA, USA). In both assays, the top chambers were filled with serum‐free medium, while the lower chamber contained medium supplemented with 10% serum as a chemoattractant. After allowing the cells to migrate for 24 h and invade for 48 h, they were fixed using methanol and subsequently stained with crystal violet. Migrated or invaded cells through the membrane were quantified under a light microscope, and the analysis was based on counting cells in three fields viewed at 100× magnification.

### 2.11 Statistical analysis

Results from the migration and invasion assays are expressed as the mean ± standard deviation (SD). Statistical analysis used Student's *t*‐test for comparisons between two groups. In examining the association between MAOB genotypic frequencies and clinicopathologic features, multivariate logistic regression models were employed to estimate odds ratios (ORs), adjusted ORs (AORs) and 95% confidence intervals (CIs). Statistical analyses were conducted using the SAS software program (vers. 9.1, 2005; SAS Institute, Cary, NC, USA). A significance threshold of *p* < 0.05 was set for all statistical evaluations.

## 3 RESULTS

### 3.1 General characteristics of PCa patients categorized based on high and low iPSA levels

In Table 1, we analysed demographic characteristics of the two iPSA groups: 334 patients with PSA levels of ≤10 ng/mL and 368 patients with PSA levels of >10 ng/mL. Compared to the ≤10 ng iPSA/mL group, the >10 ng iPSA/mL group exhibited a significantly higher frequency of developing tumours with a high Gleason grade (4 + 5) and an advanced clinical stage (T3 + 4, N1, M1). Surgical pathological observations revealed that patients with iPSA of >10 ng/mL were more prone to developing seminal vesicle, perineural and lymphovascular invasion by tumour cells, as well as exhibiting larger tumour sizes and lymph node metastasis compared to patients with iPSA of ≤10 ng/mL. According to the D'Amico risk classification for PCa, a higher proportion of patients with >10 ng iPSA/mL belonged to the high‐risk group and exhibited a higher frequency of biochemical recurrence (BR). Overall, demographic characteristics of our recruited PCa subjects with high and low iPSA were consistent with those previously reported.

**TABLE 1:** Distributions of demographic characteristics of 702 patients with prostate cancer.

Variable	PSA at diagnosis (ng/mL)		*p* value
	≤10 (*n* = 334)	>10 (*n* = 368)	
Age at diagnosis (years)			
≤65	160 (47.9%)	136 (37.0%)	***p* = 0.003**
>65	174 (52.1%)	232 (63.0%)	
Pathologic Gleason grade group			
1 + 2 + 3	304 (91.0%)	279 (75.8%)	***p* < 0.001**
4 + 5	30 (9.0%)	89 (24.2%)	
Clinical T stage			
1 + 2	314 (94.0%)	290 (78.8%)	***p* < 0.001**
3 + 4	20 (6.0%)	78 (21.2%)	
Clinical N stage			
N0	331 (99.1%)	357 (97.0%)	***p* = 0.048**
N1	3 (0.9%)	11 (3.0%)	
Clinical M stage			
M0	334 (100.0%)	357 (97.0%)	***p* = 0.001**
M1	0 (0.0%)	11 (3.0%)	
Pathologic T stage			
2	231 (69.2%)	141 (38.3%)	***p* < 0.001**
3 + 4	103 (30.8%)	227 (61.7%)	
Pathologic N stage			
N0	319 (95.5%)	324 (88.0%)	***p* < 0.001**
N1	15 (4.5%)	44 (12.0%)	
Seminal vesicle invasion			
No	303 (90.7%)	249 (67.7%)	***p* < 0.001**
Yes	31 (9.3%)	119 (32.3%)	
Perineural invasion			
No	114 (34.1%)	72 (19.6%)	***p* < 0.001**
Yes	220 (65.9%)	296 (80.4%)	
Lymphovascular invasion			
No	307 (91.9%)	283 (76.9%)	***p* < 0.001**
Yes	27 (8.1%)	85 (23.1%)	
D'Amico classification			
Low/Intermediate risk	241 (72.2%)	108 (29.3%)	***p* < 0.001**
High risk	93 (27.8%)	260 (70.7%)	
Biochemical recurrence			
No	267 (79.9%)	212 (57.6%)	***p* < 0.001**
Yes	67 (20.1%)	156 (42.4%)	

Abbreviations: M, metastasis; N, node; PSA, prostate‐specific antigen; T, tumour.

*p* < 0.05 as statistically significant values in bold.

### 3.2 Associations of MAOB genetic polymorphisms with iPSA levels in patients with PCa

We next investigated possible impacts of the four selected SNPs (rs1799836 (A/G), rs3027452 (G/A), rs6651806 (A/C) and rs6324 (G/A)) of the *MAOB* gene on iPSA levels in PCa patients. We initially analysed genotype frequencies of these SNPs in the entire recruited population. As illustrated in Table 2, the most prevalent allelic distributions for MAOB rs1799836, rs3027452, rs6651806 and rs6324 SNPs in PCa patients were the A‐, G‐, A‐ and G‐alleles, respectively. We employed AORs with 95% CIs estimated by multiple logistic regression models after adjusting for covariates between MAOB SNPs and iPSA levels. In our observations, only PCa patients carrying the MAOB rs6324 A‐allele showed a significant association with higher risks (AOR = 1.822, 95% CI = 1.065–3.118) of having >10 ng iPSA/mL, compared to those with the rs6324 G‐allele (Table 2).

**TABLE 2:** Distribution frequency of *MAOB* genotypes in 702 patients with prostate cancer.

Variable	PSA at diagnosis (ng/mL)			
	≤10 (*N* = 334) (%)	>10 (*N* = 368) (%)	OR (95% CI)	AOR (95% CI)
rs1799836				
A	275 (82.3%)	303 (82.3%)	1.000	1.000
G	59 (17.7%)	85 (17.7%)	1.000 (0.678‐1.474) *p* = 1.000	0.976 (0.624‐1.527) *p* = 0.915
rs3027452				
G	289 (86.5%)	317 (86.1%)	1.000	1.000
A	45 (13.5%)	51 (13.9%)	1.033 (0.671‐1.591) *p* = 0.882	1.082 (0.660‐1.776) *p* = 0.754
rs6651806				
A	303 (90.7%)	316 (85.9%)	1.000	1.000
C	31 (9.3%)	52 (14.1%)	1.608 (1.003‐2.578) *p* = 0.047	1.632 (0.953‐2.794) *p* = 0.074
rs6324				
G	299 (89.5%)	319 (86.7%)	1.000	1.000
A	35 (10.5%)	49 (13.3%)	1.312 (0.827‐2.082) *p* = 0.248	**1.822 (1.065–3.118)** ***p* = 0.029**

*Note*: The odds ratios (ORs) and with their 95% confidence intervals (CIs) were estimated by logistic regression models. The adjusted ORs (AORs) with their 95% CIs were estimated by multiple logistic regression models after controlling for age at diagnosis, pathologic Gleason grade group, clinical T (tumour) stage, clinical N (node) stage, clinical M (metastasis) stage, pathologic T stage, pathologic N stage, seminal vesicle invasion, perineural invasion, lymphovascular invasion, biochemical recurrence and D'Amico classification. PSA, prostate‐specific antigen.

*p* < 0.05 as statistically significant values in bold.

### 3.3 Correlation of MAOB genetic polymorphisms with clinicopathologic features of PCa patients

Next, we assessed connections of these four MAOB genetic variants with PCa clinicopathologic features, including iPSA levels, clinical TNM stages, pathologic T and N stages, tumour invasion statuses, D'Amico classification and BR (Table 3, Supplementary Table S1). Among the four MAOB loci, we observed that PCa patients who carried the rs3027452 A‐allele had a significantly higher risk of developing distal metastasis (cM1) (OR: 3.720‐fold; 95% CI: 1.068–12.959; *p* = 0.027) compared to patients carrying the wild‐type (WT) G allele (Table 3). Moreover, we stratified PCa patients into subpopulations based on iPSA levels (≤10 or >10 ng/mL) and observed that a significant impact of the rs3027452 SNP on the risk of developing distal metastasis was only evident in PCa patients with >10 ng iPSA/mL (Supplementary Table S2). Furthermore, when we stratified PCa patients into subpopulations with or without BR, we found that patients without BR harbouring the rs1799836 G‐allele showed a significantly higher risk of developing lymph node metastasis (OR: 3.508‐fold; 95% CI: 1.086–11.336; *p* = 0.026) and cancer recurrence (OR: 1.677‐fold; 95% CI: 1.045–2.692; *p* = 0.031) compared to patients harbouring the WT A‐allele (Supplementary Table S3). However, the other two MAOB SNPs, rs6651806 and rs6324, showed no significant associations with the above‐mentioned clinicopathologic features (Supplementary Table S1).

**TABLE 3:** Odds ratios (ORs) and 95% confidence intervals (CIs) of the clinical status and *MAOB* rs1799836 and rs3027452 genotypic frequencies in 702 patients with prostate cancer.

Variable	rs1799836				rs3027452			
	A (*N* = 578)	G (*N* = 124)	OR (95% CI)	*p* value	G (*N* = 606)	A (*N* = 96)	OR (95% CI)	*p* value
PSA at diagnosis (ng/mL)								
≤10	243 (42.0%)	53 (42.7%)	1.000	0.886	254 (41.9%)	42 (43.8%)	1.000	0.735
>10	335 (58.0%)	71 (57.3%)	0.972 (0.656–1.438)		352 (58.1%)	54 (56.2%)	0.928 (0.601–1.432)	
Pathologic Gleason grade group								
1 + 2	475 (82.2%)	108 (87.1%)	1.000	0.185	500 (82.5%)	83 (86.5%)	1.000	0.338
3 + 4 + 5	103 (17.8%)	16 (12.9%)	0.683 (0.388–1.204)		106 (17.5%)	13 (13.5%)	0.739 (0.397–1.375)	
Clinical T stage								
1 + 2	492 (85.1%)	112 (90.3%)	1.000	0.129	516 (85.1%)	88 (91.7%)	1.000	0.087
3 + 4	86 (14.9%)	12 (9.7%)	0.613 (0.324–1.160)		90 (14.9%)	8 (8.3%)	0.521 (0.224–1.112)	
Clinical N stage								
N0	568 (98.3%)	120 (96.8%)	1.000	0.280	595 (98.2%)	93 (96.9%)	1.000	0.394
N1	10 (1.7%)	4 (3.2%)	1.893 (0.584–6.138)		11 (1.8%)	3 (3.1%)	1.745 (0.478–6.372)	
Clinical M stage								
M0	571 (98.8%)	120 (96.8%)	1.000	0.101	599 (98.8%)	92 (95.8%)	1.000	**0.027**
M1	7 (1.2%)	4 (3.2%)	2.719 (0.784–9.435)		7 (1.2%)	4 (4.2%)	**3.720 (1.068–12.959)**	
Pathologic T stage								
2	302 (52.2%)	70 (56.5%)	1.000	0.395	319 (52.6%)	53 (55.2%)	1.000	0.639
3 + 4	276 (47.8%)	54 (43.5%)	1.186 (0.840–1.675)		287 (47.4%)	43 (44.8%)	0.902 (0.585–1.390)	
Pathologic N stage								
N0	531 (91.9%)	112 (90.3%)	1.000	0.573	557 (91.9%)	86 (89.6%)	1.000	0.444
N1	47 (8.1%)	12 (9.7%)	1.210 (0.622–2.356)		49 (8.1%)	10 (10.4%)	1.322 (0.645–2.707)	
Seminal vesicle invasion								
No	452 (78.2%)	100 (80.6%)	1.000	0.547	475 (78.4%)	77 (80.2%)	1.000	0.685
Yes	126 (21.8%)	24 (19.4%)	0.861 (0.529–1.402)		131 (21.6%)	19 (19.8%)	0.895 (0.522–1.532)	
Perineural invasion								
No	160 (27.7%)	26 (21.0%)	1.000	0.124	167 (27.6%)	19 (19.8%)	1.000	0.109
Yes	418 (72.3%)	98 (79.0%)	1.443 (0.902–2.307)		439 (72.4%)	77 (80.2%)	1.542 (0.905–2.626)	
Lymphovascular invasion								
No	487 (84.3%)	103 (83.1%)	1.000	0.742	511 (84.3%)	79 (82.3%)	1.000	0.613
Yes	91 (15.7%)	21 (16.9%)	1.091 (0.649–1.835)		95 (15.7%)	17 (17.7%)	1.157 (0.656–2.043)	
D'Amico classification								
Low/intermediate risk	288 (49.8%)	61 (49.2%)	1.000	0.898	298 (49.2%)	51 (53.1%)	1.000	0.472
High risk	290 (50.2%)	63 (50.8%)	1.026 (0.696–1.512)		308 (50.8%)	45 (46.9%)	0.854 (0.555–1.314)	
Biochemical recurrence								
No	395 (68.3%)	84 (67.7%)	1.000	0.897	414 (68.3%)	65 (67.7%)	1.000	0.905
Yes	183 (31.7%)	40 (32.3%)	1.028 (0.679–1.557)		192 (31.7%)	31 (32.3%)	1.028 (0.649–1.630)	

*Note*: ORs with their 95% CIs were estimated by logistic regression models.

Abbreviations: M, metastasis; N, node; PSA, prostate‐specific antigen; T, tumour.

*p* < 0.05 as statistically significant values in bold.

### 3.4 Downregulation of MAOB in PCa tissues correlates with tumour progression and a poor prognosis

**FIGURE 1:**
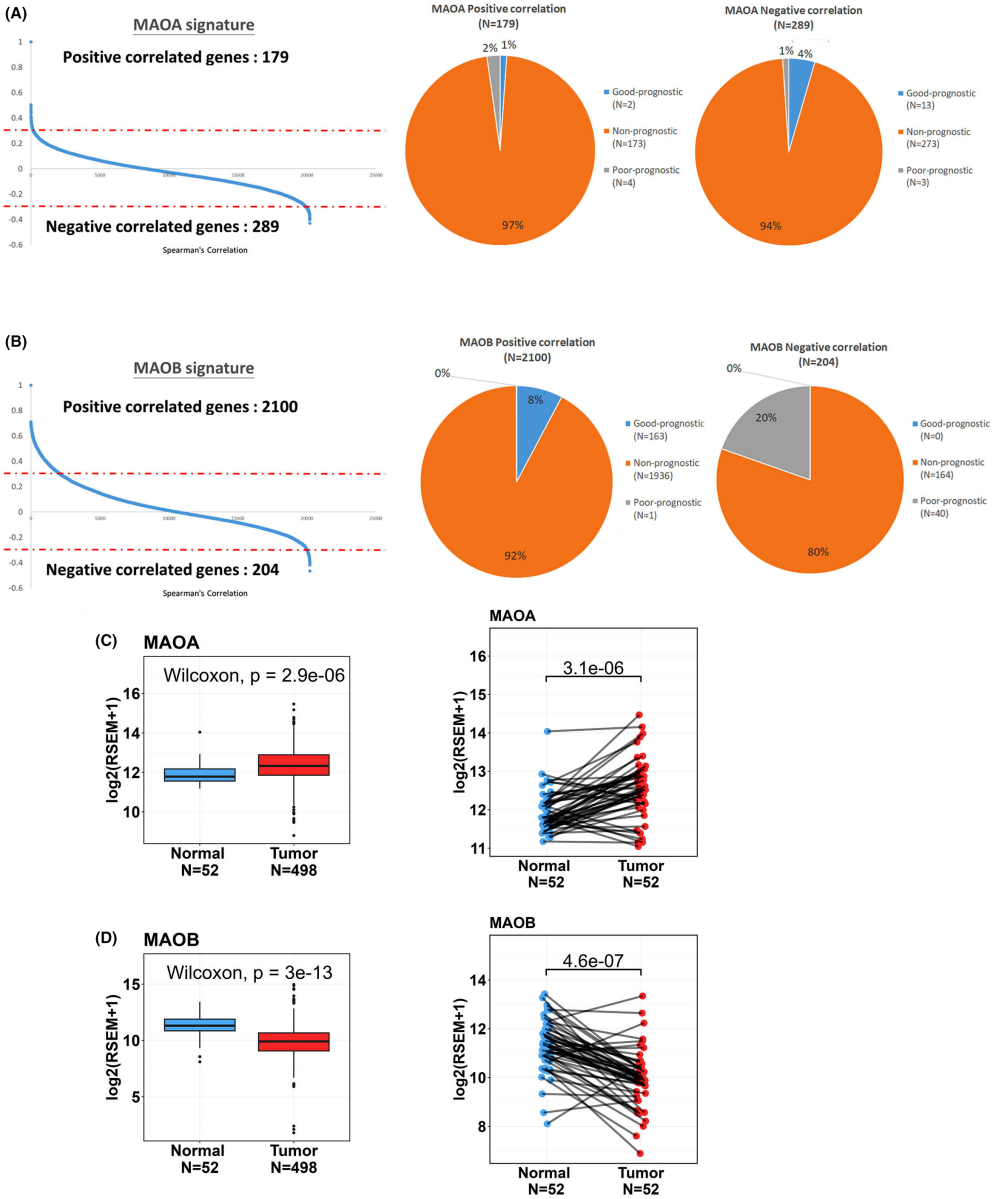
Prognostic effects of genes positively and negatively correlated with monoamine oxidases (MAOs) and their expression levels in prostate cancer (PCa) patients. (A, B) Left panel: Graphs depict correlation rankings of MAOA‐ and MAOB‐associated genes, with the red dashed lines indicating the threshold used to define correlated candidates. Genes with Spearman correlation coefficients of >0.3 or <−0.3 were considered correlated with MAOA or MAOB gene expressions. Right panel: Pie charts illustrate percentages of MAOA‐ or MAOB‐associated gene candidates that have prognostic prediction value. Prognostic effects of MAOA‐ or MAOB‐correlated genes were assessed through a Cox regression analysis in patients with PCa from The Cancer Genome Atlas (TCGA)‐prostate adenocarcinoma (PRAD) data set. Genes with a hazard ratio (HR) of >1 and *p* < 0.05 were categorized as poor prognostic genes, while genes with an HR of <1 and *p* < 0.05 are classified as good prognostic genes. Gene candidates that did not meet these criteria were considered non‐prognostic. (C, D) Left panel: *MAOA* and *MAOB* gene expression levels in unpaired normal and tumour tissues are presented using data from TCGA‐PRAD data set. Right panel: MAOA and MAOB expression levels were analysed in 52 matched PCa tissues and their corresponding normal tissues.

MAOA expression was reported to be correlated with the progression and poor prognosis of PCa.^31^ Herein, we first compared the prognostic values of both MAOA and MAOB in PCa using an online TCGA‐PRAD cohort. The correlation analysis of MAO expression and genes in PCa patients revealed 468 MAOA‐correlated genes, including 179 with a positive correlation (Spearman's correlation coefficient >0.3) and 289 with a negative correlation (Spearman's correlation coefficient <0.3) (Figure 1A, left panel). On the other hand, we identified 2304 MAOB‐correlated genes, consisting of 2100 with a positive correlation and 204 with a negative correlation (Figure 1B, left panel). To assess the prognostic significance of genes correlated with MAOs, we conducted univariate Cox proportional hazard analysis on all genes in TCGA‐PRAD cohort. Hazard ratios (HRs) were used to evaluate relationship between genes and clinical outcomes, with an HR of >1 indicating poor prognostic markers and an HR of <1 indicating good prognostic markers. Among genes correlated with MAOA, approximately 1% of MAOA positively correlated genes were associated with a good prognosis (HR <1; *p* < 0.05), while 2% of MAOA positively correlated genes represented poor prognostic markers (HR >1; *p* < 0.05). Additionally, we observed that 4% of MAOA negatively correlated genes were linked to a good prognosis (HR <1; *p* < 0.05), and only 1% of MAOA negatively correlated genes were linked to a poor prognosis (HR >1; *p* < 0.05) (Figure 1A, right panel). Conversely, all of the MAOB positively correlated genes were indicative of good prognostic markers, while all of the MAOB negatively correlated genes were indicative of poor prognostic markers (Figure 1B, right panel). From TCGA‐PRAD data set, we also observed that MAOA expression levels were significantly higher in PCa tissues compared to both noncancerous tissues (Figure 1C, left panel) and corresponding matched normal tissues (Figure 1C, right panel). Conversely, MAOB expression levels were notably lower in PCa tissues compared to noncancerous tissues (Figure 1D, left panel) and corresponding matched normal tissues (Figure 1D, right panel).

**FIGURE 2:**
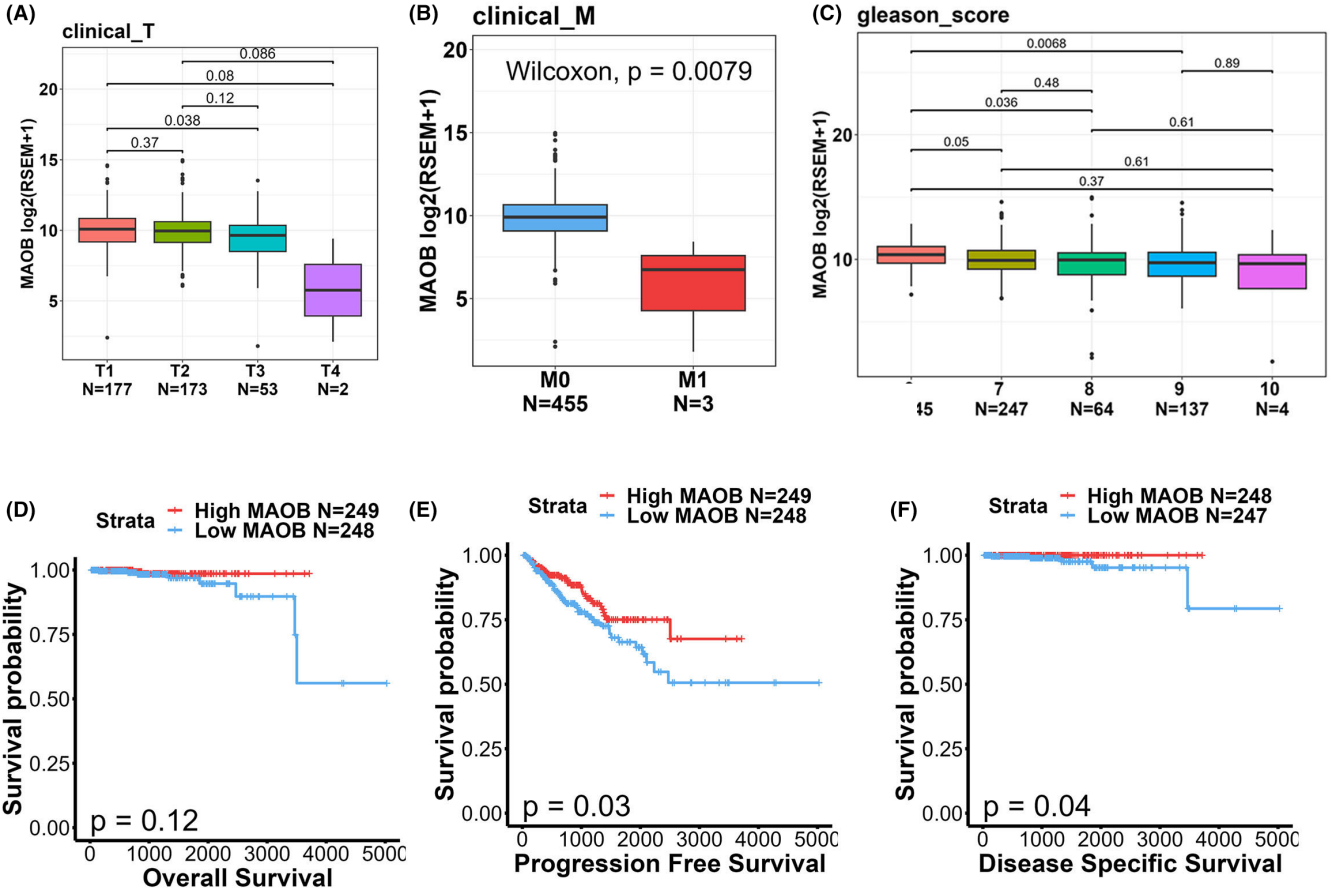
Associations between monoamine oxidase B (MAOB) and clinical features in prostate cancer (PCa) patients. (A–C) Comparison of *MAOB* gene expression levels in PCa from The Cancer Genome Atlas (TCGA)‐prostate adenocarcinoma (PRAD) data set based on clinical T stage (A), clinical M stage (B) and Gleason scores (C). (D–F) Kaplan–Meier curves illustrating overall survival (D), progression‐free survival (E) and disease‐specific survival (F) in PCa patients, stratified by high or low MAOB expression. The *p* value indicates a comparison between patients with high MAOB expression and low MAOB expression.

To delve deeper into associations between MAOB expression levels and clinicopathological features and prognoses of PCa patients, we noted that expression levels of MAOB transcripts were diminished in individuals with advanced clinical T stages (Figure 2A), distal metastasis (Figure 2B) and high Gleason scores (Figure 2C). Moreover, a Kaplan–Meier plot revealed that PCa patients with MAOB^low^ tumours had shorter OS (Figure 2D), PFS (Figure 2E) and DSS (Figure 2F) times compared to those with MAOB^high^ tumours. Collectively, while MAOA was reported to be an oncogene, our findings from gene correlation and expression analyses suggest that MAOB may potentially function as a tumour‐suppressive gene in PCa.

### 3.5 MAOB expression suppresses proliferation, migration and invasion of PCa cells

**FIGURE 3:**
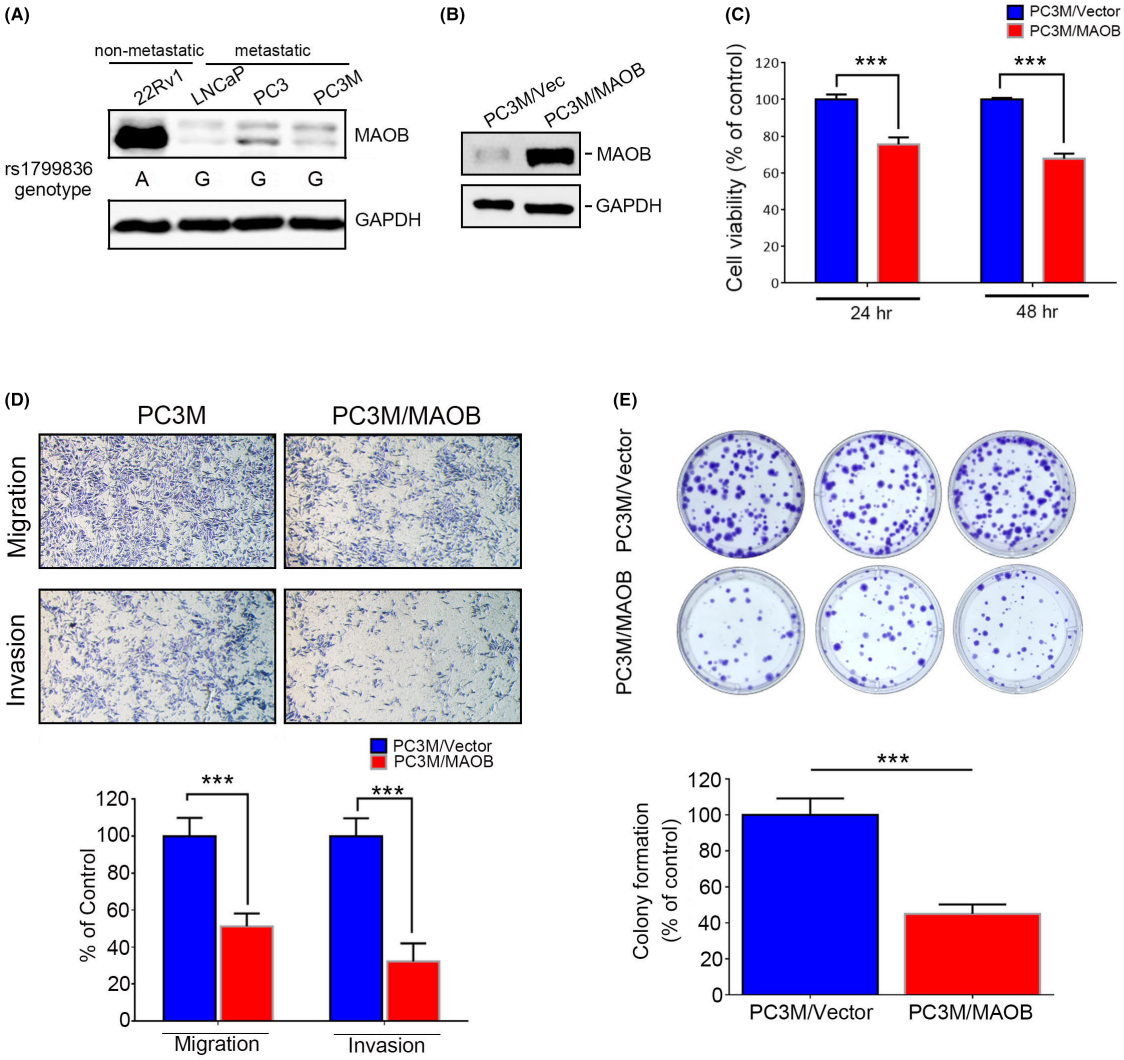
Overexpression of monoamine oxidase B (MAOB) suppresses proliferation, colony formation, migration, and invasion of prostate cancer (PCa) cells. (A) Genotypes of MAOB rs1799836 and protein levels of MAOB were respectively determined by TaqMan SNP genotyping and western blot assays in a set of PCa cell lines including non‐metastatic cells (22Rv1) and metastatic cells (LNCaP, PC3 and PC3M). (B) Western blot analysis of MAOB expressions in PC3M cells after transducing an MAOB‐expressing vector or control vector. (C) PC3M cells overexpressing MAOB were cultured in regular medium for 24 and 48 h. Changes in cell viability were determined by MTS assays. Data are presented as the mean ± standard deviation (SD) from three independent experiments. *** *p* < 0.001 compared to that of the vector control group. (D) Transwell migration and Matrigel invasion assays were performed with PC3M cells which were transduced with a MAOB‐expressing vector or control vector. (E) Effects of MAOB overexpression on the long‐term growth (14 days) of PC3M cells were determined with a colony‐formation assay. (D, E) Upper panel: Representative photomicrographs. Lower panel: Data are presented as the mean ± SD of three independent experiments. ***p < 0.001 compared to that of the vector control group.

**FIGURE 4:**
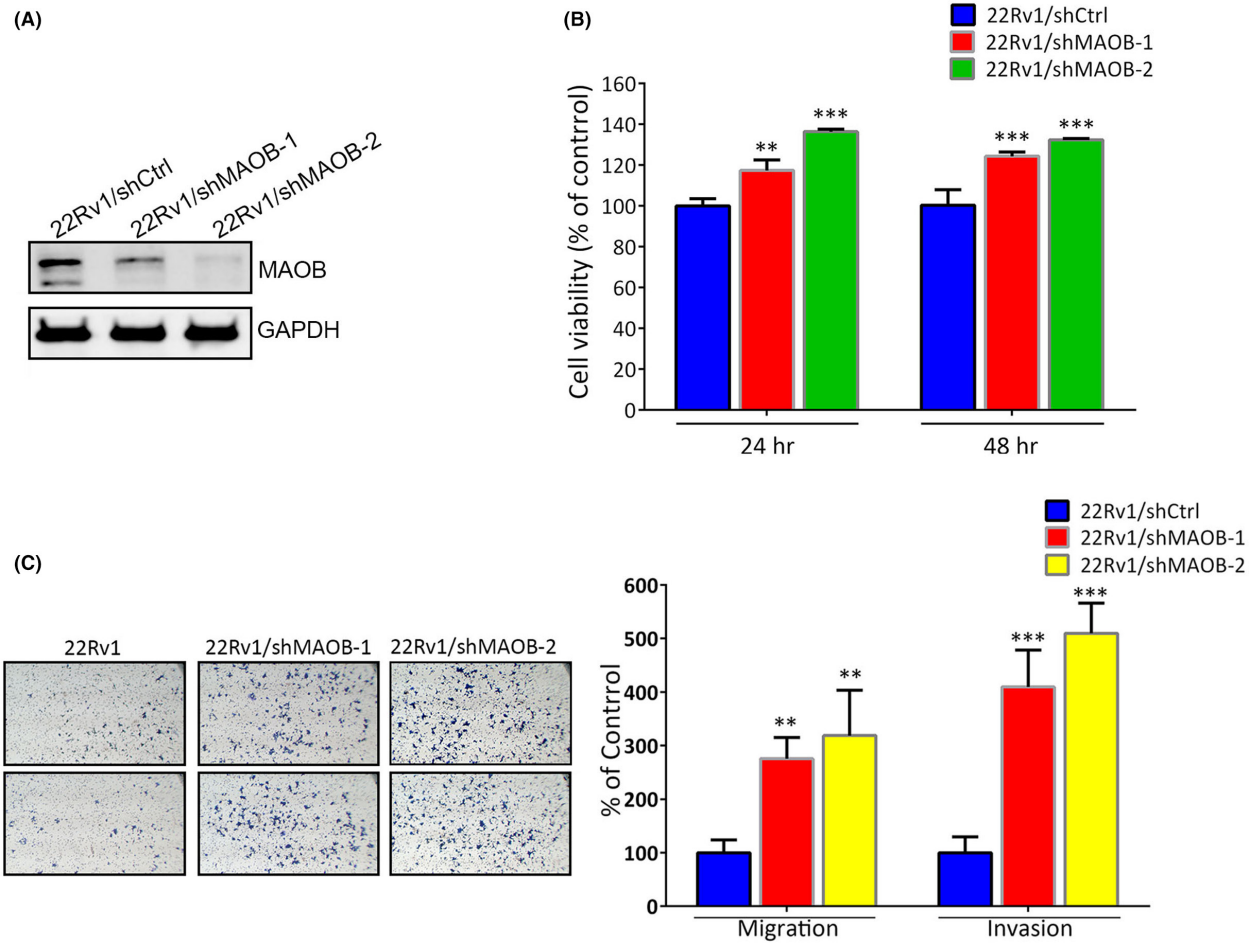
Knockdown of monoamine oxidase B (MAOB) enhances proliferation, migration and invasion of prostate cancer (PCa) cells. 22Rv1 cells were infected with a lentivirus that carried specific MAOB short hairpin (sh)RNAs or shcontrol (shCtrl) and subsequently subjected to western blot (A), MTS (B) migration and Matrigel invasion (C) assays. Left panel of (C): Representative photomicrographs. Data are presented as the mean ± SD of three independent experiments. ** *p* < 0.01, *** *p* < 0.001 compared to that of the shCtrl group.

In our in vitro study, we initially assessed MAOB expression levels and the MAOB rs1799836 genotype in a panel of PCa cell lines, including 22Rv1 (non‐metastatic) and LNCaP, PC3 and PC3M (metastatic cells), using western blotting and a real‐time PCR. Our observations revealed that 22Rv1 cells exhibited higher MAOB protein levels compared to LNCaP, PC3 and PC3M cells. Additionally, 22Rv1 cells carried the A‐allele of rs1799836, whereas LNCaP, PC3 and PC3M cells carried the G‐allele (Figure 3A). Subsequently, to investigate whether MAOB plays a role in modulating the progression of PCa cells, we conducted MAOB overexpression experiments in PC3M cells (Figure 3B). Our findings indicated that regardless of the proliferative, migratory or invasive abilities of PC3M cells, all were significantly reduced by MAOB overexpression compared to control cells (Figure 3C,D). Notably, the impact of MAOB was more pronounced on the motility than the proliferation of PCa cells during short‐term MAOB overexpression (24–48 h). We further tested the long‐term growth inhibitory effect of MAOB using a colony‐forming assay and found that long‐term overexpression (14 days) of MAOB significantly suppressed the colony‐forming abilities of PC3M cells (Figure 3E). In contrast to MAOB overexpression, we employed a lentiviral‐based RNA knockdown approach to establish stable MAOB knockdown in 22Rv1 cells (Figure 4A). We observed a significant increase in 22Rv1 cell proliferation, migration and invasion upon the knockdown of MAOB using two specific MAOB‐targeting shRNAs (Figure 4B,C). In conclusion, these in vitro cell studies provide further validation of the tumour‐suppressive role of MAOB in PCa progression. Additionally, our results suggest that MAOB SNPs might influence PCa progression by affecting MAOB expression.

### 3.6 MAOB‐modulated PCa progression is dependent on its enzyme activity

**FIGURE 5:**
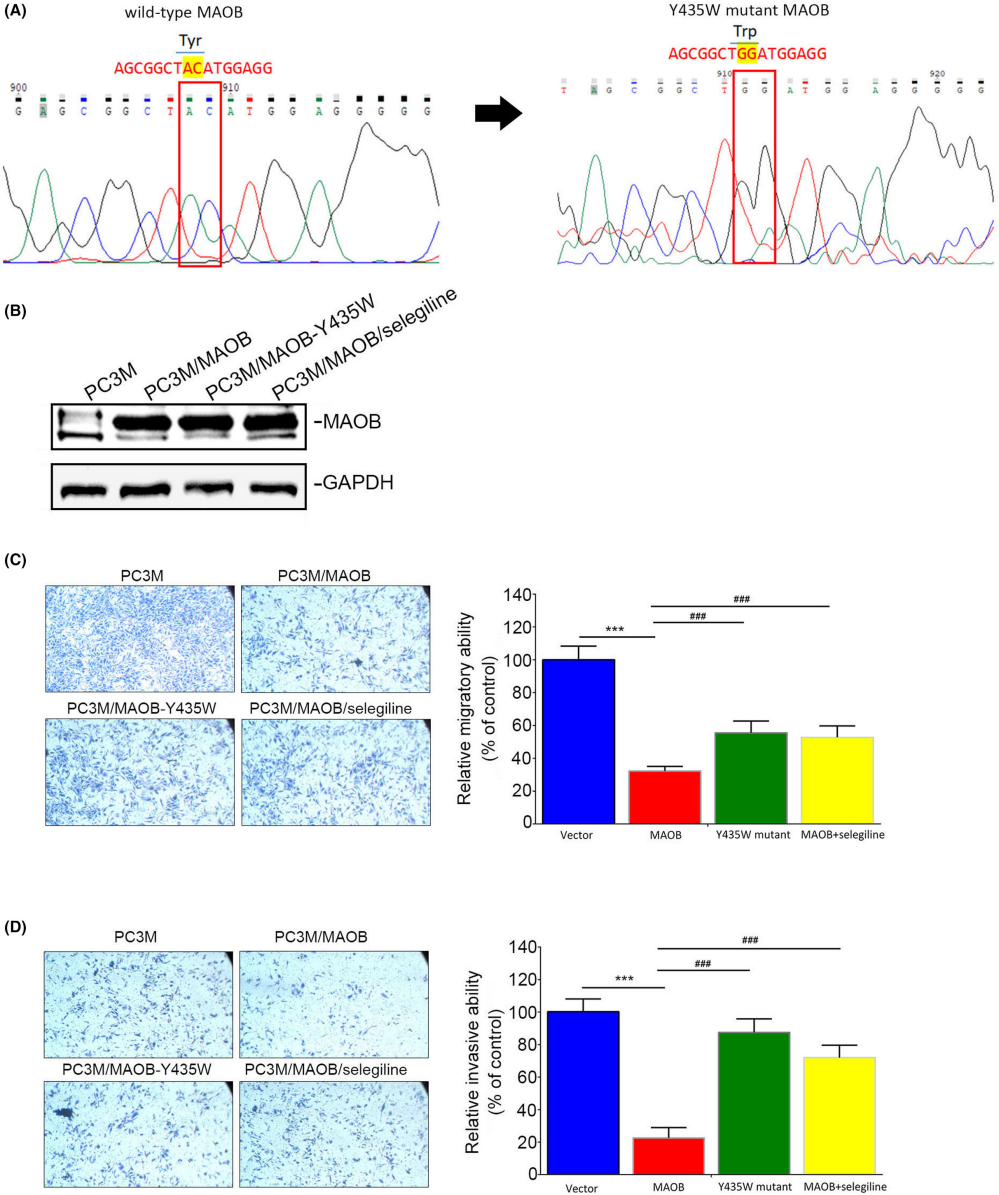
Modulation of prostate cancer (PCa) cell motility by monoamine oxidase B (MAOB) depends on its catalytic activity. (A) Sequence analysis of the Y435W MAOB mutant construct we generated. (B) Western blot analysis of MAOB expression in PC3M cells transduced with either MAOB or Y435W MAOB mutant‐expressing vectors, as well as in PC3M/MAOB cells treated with 10 μM of selegiline, a MAOB‐selective inhibitor. (C, D) Transwell migration (C) and Matrigel invasion (D) assays were conducted in PC3M cells transduced with either MAOB or Y435W MAOB mutant‐expressing vectors, as well as in PC3M/MAOB cells treated with 10 μM of the MAOB‐selective inhibitor, selegiline. The left panel shows representative photomicrographs, and the right panel presents data as the mean ± SD from three independent experiments. ***p < 0.001 compared to the vector control group. ^###^ *p* < 0.001 compared to the MAOB overexpression group.

Tyr‐435 of MAOB was reported to play a crucial role in regulating its catalytic activity.^28^ In this study, our objective was to generate a Y435W MAOB mutant construct (Figure 5A), which was reported to reduce MAOB catalytic activity.^32^ We independently overexpressed both the WT MAOB and Y435W MAOB mutant in PC3M cells (Figure 5B) to explore functional differences between the WT MAOB and Y435W mutant. Compared to MAOB, overexpression of the Y435W mutant showed a reduced inhibitory effect on the migratory (Figure 5C) and invasive (Figure 5D) capabilities of PC3M cells. Furthermore, we investigated the rescue effect of the MAOB‐selective inhibitor, selegiline, in PC3M/MAOB cells. The results showed that selegiline treatment significantly reversed the inhibitory effects on migration and invasion induced by MAOB overexpression in PC3M cells (Figure 5C,D). Taken together, these findings suggest that MAOB enzyme activity is crucial for its anticancer activity in PCa cells.

## 4 DISCUSSION

The MAOA neurotransmitter catabolic enzyme was previously reported to be upregulated in PCa and to exert an oncogenic role in promoting tumorigenesis and cancer metastasis.^20^ However, the role of the other MAO isoenzyme, MAOB, in the progression of PCa remains unclear. In the current study, we observed that all genes positively correlated with MAOB were indicative of good prognostic markers, while those negatively correlated were indicative of poor prognostic markers in PCa patients. Furthermore, we found that MAOB was downregulated in PCa tissues, was inversely correlated with the Gleason score, cT stage and metastasis, and was associated with favourable clinical outcomes. In vitro experiments revealed that MAOB overexpression in PCa cells led to suppression of both proliferation and motility. Collectively, these results strongly support the notion that MAOB plays an opposing role to MAOA in modulating PCa progression. While the contrasting roles of MAOA and MAOB in PCa progression remain unexplained, we speculated that distinct substrates may modulate the tumour‐suppressive activities of MAOB and oncoprotein activities of MAOA. Resolving this discrepancy will require further studies.

Genetic variants in the *MAOB* gene were linked to alterations in MAOB enzyme activity or gene expression, showing correlations with several neurodegenerative diseases. For instance, individuals with the MAOB rs1799836 G‐allele in Parkinson's disease have a higher likelihood of developing dyskinesia compared to those with the A‐allele, potentially due to the G‐allele's association with reduced MAOB activity in the brain.^24^ Balciuniene et al. also indicated that lower MAOB enzyme activity was observed in human brain samples from subjects with the rs1799836 G‐allele compared to the A‐allele.^33^ In male patients with schizophrenia, the presence of the MAOB rs1799836 A‐allele, presumably linked to elevated MAOB activity, was associated with a greater severity of negative symptoms.^23^ Beyond rs1799836, another intronic SNP, the rs3027452 A‐allele, was associated with the lowest transcription rates in the central nervous system. This was expected to be correlated with reduced MAOB activity and to coincide with an elevation in serotonin concentrations.^34^ Additionally, apart from its association with neurodegenerative diseases, the rs3027452 A‐allele was reported to be linked to lower MAOB activity and a correlation with blood pressure in obese hypogonadic patients with hypertension.^26^

Relationships of MAOB SNPs with cancer have been infrequently discussed. Our current data revealed that among PCa patients, those carrying the MAOB rs6324 A‐allele exhibited a significant association with higher risks of having iPSA levels of >10 ng/mL, compared to individuals with the rs6324 G‐allele. Moreover, PCa patients with a mutant A‐allele of rs3027452 had a significantly elevated risk of developing distal metastasis, particularly in the subpopulation with high iPSA levels (>10 ng/mL). Furthermore, our findings indicate that patients without BR harbouring the rs1799836 G‐allele showed a significantly increased risk of developing lymph node metastasis. According to prior research and our clinical and in vitro studies described above, MAOB may function as a tumour suppressor in PCa. The correlation between the rs3027452 A‐allele and rs1799836 G‐allele with a higher risk of PCa metastasis may be attributed to lower enzyme activity of MAOB. Actually, our present study demonstrated that metastatic PCa cells (LNCaP, PC3 and PC3M) harbouring the rs1799836 G‐allele expressed lower levels of MAOB than did non‐metastatic PCa cells (22Rv1) harbouring the A‐allele. Furthermore, we showed that the MAOB‐suppressed motility of PCa cells was dependent on its enzyme activity. These results suggest that the impact of MAOB SNPs on enzyme activity might be associated with changes in *MAOB* gene expression.

Both rs3027452 and rs1799836 SNPs are situated within intron regions of MAOB. While polymorphisms in intronic regions traditionally do not alter the protein sequence, emerging evidence suggests that such variations can induce splicing abnormalities, potentially influencing translation and contributing to human diseases, including cancers.^35^ For instance, the MAOB rs1799836 A‐allele is thought to enhance the removal of MAOB intron 13 in Parkinson's disease patients. This removal of non‐coding sequences might act as a rate‐limiting factor in MAOB mRNA formation, leading to increased protein levels and enzymatic activity.^36^ Additionally, intronic sequences harbour numerous cis‐acting regulatory elements (CREs), such as transcription factors, enhancers and silencers, which can either positively or negatively regulate gene expressions.^37^ As an example, the rs12343867 T > C SNP in intron 14 of Janus‐activated kinase 2 (JAK2) is associated with myeloproliferative neoplasms, functioning as a transcriptional repressor.^38^ Indeed, a previous report suggested that the presence of a CRE in linkage disequilibrium (LD) with MAOB rs1799836 altered MAOB protein expression and activity.^39^ Apart from CREs, many long non‐coding (lnc)RNAs are located within intronic sequences and are reported to regulate expressions of their corresponding host genes.^37^ Whether the rs3027452 and rs1799836 SNPs are situated in any intronic lncRNA regions remains unknown. Further investigation is necessary to clarify the specific impacts of these SNPs on *MAOB* gene expression.

However, this study possesses certain limitations that warrant discussion. Specifically, our research solely involved participants from the Taiwanese population. Inclusion of diverse ethnic populations in future investigations would facilitate cross‐comparisons and validation of findings across different racial groups. Additionally, for a more comprehensive exploration of the impact of MAOB SNPs on both MAOB expression and enzyme activity, simultaneous collection of mRNA, DNA and protein from the same samples should be considered in future research. Furthermore, it is essential to recognize that our study focused exclusively on the association of MAOB SNPs with PCa, and further exploration into the effects of genetic variations in its isoenzyme, MAOA, is warranted.

In summary, our study is the first to investigate the diverse allelic effects of MAOB SNPs in a Taiwanese population, revealing an impact on the metastasis status of PCa. Furthermore, we demonstrated that the tumour‐suppressive role of MAOB was dependent on its enzyme activity in PCa cells. In a clinical context, we observed a favourable prognostic effect of MAOB in PCa clinical samples. Our findings suggest that genetic variations and downregulation of MAOB may play potential roles in PCa development, and the MAOB rs3027452 and rs1799836 polymorphisms may serve as pivotal markers for predicting PCa tumour metastasis and prognosis.

## AUTHOR CONTRIBUTIONS

**Hsiang‐Ching Huang:** Conceptualization (equal); data curation (equal); writing – original draft (equal). **Yi‐Hsien Hsieh:** Conceptualization (equal); data curation (equal). **Chi‐Hao Hsiao:** Conceptualization (equal). **Chia‐Yen Lin:** Data curation (equal); resources (equal). **Shian‐Shiang Wang:** Data curation (equal); resources (equal). **Kuo‐Hao Ho:** Data curation (equal); software (equal). **Lun‐Ching Chang:** Software (equal). **Huei‐Mei Huang:** Conceptualization (equal). **Shun‐Fa Yang:** Conceptualization (equal); methodology (equal); writing – original draft (equal). **Ming‐Hsien Chien:** Conceptualization (equal); funding acquisition (equal); software (equal); writing – original draft (equal); writing – review and editing (equal).

## CONFLICT OF INTEREST STATEMENT

The authors declare no conflicts of interest related to this study.

## Supporting information


Supplementary Table S1.

## Data Availability

The data used to support the findings of this study are available from the corresponding author upon reasonable request.
