# Supplementary material for: MAOB expression correlates with a favourable prognosis in prostate cancer, and its genetic variants are associated with the metastasis of the disease
Source: J Cell Mol Med. 2024 Mar 23;28(8):e18229. doi: 10.1111/jcmm.18229 (PMC10960177; doi:10.1111/jcmm.18229)
Supplement: Supplementary file 1 — Supplementary Table S1. [file JCMM-28-e18229-s001.docx]

**Supplementary table 1.** Odds ratios (ORs) and 95% confidence intervals (CIs) of the clinical status and *MAOB* rs6651806 and rs6324 genotypic frequencies in 702 patients with prostate cancer

| **Variable** | **rs6651806** | | | | **rs6324** | | | |
| --- | --- | --- | --- | --- | --- | --- | --- | --- |
|  | **A**  **(*N*=619)** | **C**  **(*N*=83)** | **OR (95% CI)** | ***p* value** | **G**  **(*N*=618)** | **A**  **(*N*=84)** | **OR (95% CI)** | ***p* value** |
| **PSA at diagnosis (ng/mL)** |  |  |  |  |  |  |  |  |
| <10 | 258 (41.7%) | 38 (45.8%) | 1.000 | 0.477 | 254 (41.1%) | 42 (50.0%) | 1.000 | 0.121 |
| >10 | 361 (58.3%) | 45 (54.2%) | 0.846 (0.534~1.341) |  | 364 (58.9%) | 42 (50.0%) | 0.698 (0.442~1.102) |  |
| **Pathologic Gleason grade group** |  |  |  |  |  |  |  |  |
| 1+2 | 514 (83.0%) | 69 (83.1%) | 1.000 | 0.983 | 513 (83.0%) | 70 (83.3%) | 1.000 | 0.941 |
| 3+4+5 | 105 (17.0%) | 14 (16.9%) | 0.993 (0.539~1.831) |  | 105 (17.0%) | 14 (16.7%) | 0.977 (0.530~1.800) |  |
| **Clinical T stage** |  |  |  |  |  |  |  |  |
| 1+2 | 535 (86.4%) | 69 (83.1%) | 1.000 | 0.416 | 530 (85.8%) | 74 (88.1%) | 1.000 | 0.562 |
| 3+4 | 84 (13.6%) | 14 (16.9%) | 1.292 (0.696~2.399) |  | 88 (14.2%) | 10 (11.9%) | 0.814 (0.405~1.635) |  |
| **Clinical N stage** |  |  |  |  |  |  |  |  |
| N0 | 607 (98.1%) | 81 (97.6%) | 1.000 | 0773 | 604 (97.7%) | 84 (100.0%) | 1.000 | 0.163 |
| N1 | 12 (1.9%) | 2 (2.4%) | 1.249 (0.275~5.681) |  | 14 (2.3%) | 0 (0.0%) | --- |  |
| **Clinical M stage** |  |  |  |  |  |  |  |  |
| M0 | 609 (98.4%) | 82 (98.8%) | 1.000 | 0.777 | 608 (98.4%) | 83 (98.8%) | 1.000 | 0.767 |
| M1 | 10 (1.6%) | 1 (1.2%) | 0.743 (0.094~5.877) |  | 10 (1.6%) | 1 (1.2%) | 0.733 (0.093~5.796) |  |
| **Pathologic T stage** |  |  |  |  |  |  |  |  |
| 2 | 326 (52.7%) | 46 (55.4%) | 1.000 | 0.637 | 323 (52.3%) | 49 (58.3%) | 1.000 | 0.296 |
| 3+4 | 293 (47.3%) | 37 (44.6%) | 0.895 (0.565~1.419) |  | 295 (47.7%) | 35 (41.7%) | 0.782 (0.493~1.241) |  |
| **Pathologic N stage** |  |  |  |  |  |  |  |  |
| N0 | 567 (91.6%) | 76 (91.6%) | 1.000 | 0.992 | 563 (91.1%) | 80 (95.2%) | 1.000 | 0.200 |
| N1 | 52 (8.4%) | 7 (8.4%) | 1.004 (0.440~2.291) |  | 55 (8.9%) | 4 (4.8%) | 0.512 (0.181~1.450) |  |
| **Seminal vesicle invasion** |  |  |  |  |  |  |  |  |
| No | 448 (78.8%) | 64 (77.1%) | 1.000 | 0.718 | 485 (78.5%) | 67 (79.8%) | 1.000 | 0.788 |
| Yes | 131 (21.2%) | 19 (22.9%) | 1.106 (0.640~1.911) |  | 133 (21.5%) | 17 (20.2%) | 0.925 (0.525~1.629) |  |
| **Perineural invasion** |  |  |  |  |  |  |  |  |
| No | 164 (26.5%) | 22 (26.5%) | 1.000 | 0.998 | 159 (25.7%) | 27 (32.1%) | 1.000 | 0.211 |
| Yes | 455 (73.5%) | 61 (73.5%) | 0.999 (0.595~1.679) |  | 459 (74.3%) | 57 (67.9%) | 0.731 (0.447~1.196) |  |
| **Lymphovascular invasion** |  |  |  |  |  |  |  |  |
| No | 521 (84.2%) | 69 (83.1%) | 1.000 | 0.809 | 517 (83.7%) | 73 (86.9%) | 1.000 | 0.446 |
| Yes | 99 (15.8%) | 14 (16.9%) | 1.079 (0.584~1.993) |  | 101 (16.3%) | 11 (13.1%) | 0.771 (0.395~1.506) |  |
| **D’Amico classification** |  |  |  |  |  |  |  |  |
| Low/Intermediate risk | 315 (50.9%) | 34 (41.0%) | 1.000 | 0.089 | 303 (49.0%) | 46 (54.8%) | 1.000 | 0.324 |
| High risk | 304 (49.1%) | 49 (59.0%) | 1.493 (0.938~2.377) |  | 315 (51.0%) | 38 (45.2%) | 0.795 (0.503~1.256) |  |
| **Biochemical recurrence** |  |  |  |  |  |  |  |  |
| No | 418 (67.5%) | 61 (73.5%) | 1.000 | 0.273 | 414 (67.0%) | 65 (77.4%) | 1.000 | 0.055 |
| Yes | 201 (32.5%) | 22 (26.5%) | 0.750 (0.448~1.256) |  | 204 (33.0%) | 19 (22.6%) | 0.593 (0.346~1.016) |  |

ORs with their 95% CIs were estimated by logistic regression models. PSA, prostate-specific antigen; T, tumor; N, node; M, metastasis.

**Supplementary table 2.** Odds ratios (ORs) and 95% confidence intervals (CIs) of the clinical status and *MAOB* rs3027452 genotypic frequencies in 368 prostate cancer patients with a prostate-specific antigen level of >10 ng/mL

| **Variable** | **Genotypic frequencies** | | | |
| --- | --- | --- | --- | --- |
| **rs3027452** | **G**  **(*N*=317)** | **A**  **(*N*=51)** | **OR (95% CI)** | ***p* value** |
| **Pathologic Gleason grade group** |  |  |  |  |
| 1+2 | 238 (75.1%) | 41 (80.4%) | 1.000 | 0.411 |
| 3+4+5 | 79 (24.9%) | 10 (19.6%) | 0.735 (0.352~1.535) |  |
| **Clinical T stage** |  |  |  |  |
| 1+2 | 246 (77.6%) | 44 (86.3%) | 1.000 | 0.160 |
| 3+4 | 71 (22.4%) | 7 (13.7%) | 0.551 (0.238~1.277) |  |
| **Clinical N stage** |  |  |  |  |
| N0 | 307 (96.8%) | 50 (98.0%) | 1.000 | 0.642 |
| N1 | 10 (3.2%) | 1 (2.0%) | 0.614 (0.077~4.901) |  |
| **Clinical M stage** |  |  |  |  |
| M0 | 310 (97.8%) | 47 (92.2%) | 1.000 | **0.028*** |
| M1 | 7 (2.2%) | 4 (7.8%) | **3.769 (1.062~13.370)** |  |
| **Pathologic T stage** |  |  |  |  |
| 2 | 121 (38.2%) | 20 (39.2%) | 1.000 | 0.887 |
| 3+4 | 196 (61.8%) | 31 (60.8%) | 0.957 (0.522~1.754) |  |
| **Pathologic N stage** |  |  |  |  |
| N0 | 280 (88.3%) | 44 (86.3%) | 1.000 | 0.675 |
| N1 | 37 (11.7%) | 7 (13.7%) | 1.204 (0.505~2.868) |  |
| **Seminal vesicle invasion** |  |  |  |  |
| No | 215 (67.8%) | 34 (66.7%) | 1.000 | 0.870 |
| Yes | 102 (32.2%) | 17 (33.3%) | 1.054 (0.562~1.975) |  |
| **Perineural invasion** |  |  |  |  |
| No | 64 (20.2%) | 8 (15.7%) | 1.000 | 0.452 |
| Yes | 253 (79.8%) | 43 (84.3%) | 1.360 (0.609~3.035) |  |
| **Lymphovascular invasion** |  |  |  |  |
| No | 244 (77.0%) | 39 (76.5%) | 1.000 | 0.937 |
| Yes | 73 (23.0%) | 12 (23.5%) | 1.028 (0.512~2.067) |  |
| **D’Amico classification** |  |  |  |  |
| Low/Intermediate risk | 91 (28.7%) | 17 (33.3%) | 1.000 | 0.501 |
| High risk | 226 (71.3%) | 34 (66.7%) | 0.805 (0.428~1.514) |  |
| **Biochemical recurrence** |  |  |  |  |
| No | 182 (57.4%) | 30 (58.8%) | 1.000 | 0.850 |
| Yes | 135 (42.6%) | 21 (41.2%) | 0.944 (0.518~1.720) |  |

ORs with their 95% CIs were estimated by logistic regression models. T, tumor; N, node; M, metastasis.

* *p*<0.05 as statistically significant.

**Supplementary table 3.** Odds ratios (ORs) and 95% confidence intervals (CIs) of the clinical status and *MAOB* rs1799836 genotypic frequencies in 479 prostate cancer patients without biochemical recurrence

| **Variable** | **Genotypic frequencies** | | | |
| --- | --- | --- | --- | --- |
| **rs1799836** | **A**  **(*N*=395)** | **G**  **(*N*=84)** | **OR (95% CI)** | ***p* value** |
| **PSA at diagnosis (ng/mL)** |  |  |  |  |
| <10 | 222 (56.2%) | 45 (53.6%) | 1.000 | 0.659 |
| >10 | 173 (43.8%) | 39 (46.4%) | 1.112 (0.693~1.784) |  |
| **Pathologic Gleason grade group** |  |  |  |  |
| 1+2 | 361 (91.4%) | 78 (92.9%) | 1.000 | 0.659 |
| 3+4+5 | 34 (8.6%) | 6 (7.1%) | 0.817 (0.331~2.012) |  |
| **Clinical T stage** |  |  |  |  |
| 1+2 | 361 (91.4%) | 76 (90.5%) | 1.000 | 0.787 |
| 3+4 | 34 (8.6%) | 8 (9.5%) | 1.118 (0.498~2.510) |  |
| **Clinical N stage** |  |  |  |  |
| N0 | 390 (98.7%) | 82 (97.6%) | 1.000 | 0.439 |
| N1 | 5 (1.3%) | 2 (2.4%) | 1.902 (0.363~9.976) |  |
| **Clinical M stage** |  |  |  |  |
| M0 | 392 (99.2%) | 83 (98.8%) | 1.000 | 0.693 |
| M1 | 3 (0.8%) | 1 (1.2%) | 1.574 (0.162~15.323) |  |
| **Pathologic T stage** |  |  |  |  |
| 2 | 264 (66.8%) | 55 (65.5%) | 1.000 | 0.810 |
| 3+4 | 131 (33.2%) | 29 (34.5%) | 1.063 (0.647~1.745) |  |
| **Pathologic N stage** |  |  |  |  |
| N0 | 388 (98.2%) | 79 (94.0%) | 1.000 | **0.026*** |
| N1 | 7 (1.8%) | 5 (6.0%) | **3.508 (1.086~11.336)** |  |
| **Seminal vesicle invasion** |  |  |  |  |
| No | 358 (90.6%) | 76 (90.5%) | 1.000 | 0.964 |
| Yes | 37 (9.4%) | 8 (9.5%) | 1.018 (0.456~2.274) |  |
| **Perineural invasion** |  |  |  |  |
| No | 146 (37.0%) | 22 (26.2%) | 1.000 | 0.060 |
| Yes | 249 (63.0%) | 62 (73.8%) | 1.652 (0.975~2.800) |  |
| **Lymphovascular invasion** |  |  |  |  |
| No | 368 (93.2%) | 77 (91.7%) | 1.000 | 0.627 |
| Yes | 27 (6.8%) | 7 (8.3%) | 1.239 (0.521~2.948) |  |
| **D’Amico classification** |  |  |  |  |
| Low/Intermediate risk | 234 (59.2%) | 39 (46.4%) | 1.000 | **0.031*** |
| High risk | 161 (40.8%) | 45 (53.6%) | **1.677 (1.045~2.692)** |  |

ORs with their 95% CIs were estimated by logistic regression models. PSA, prostate-specific antigen; T, tumor; N, node; M, metastasis.

* *p*<0.05 as statistically significant.
